# A Review of Sensors and Biosensors Modified with Conducting Polymers and Molecularly Imprinted Polymers Used in Electrochemical Detection of Amino Acids: Phenylalanine, Tyrosine, and Tryptophan

**DOI:** 10.3390/ijms23031218

**Published:** 2022-01-22

**Authors:** Ancuța Dinu, Constantin Apetrei

**Affiliations:** Department of Chemistry, Physics and Environment, Faculty of Sciences and Environment, “Dunărea de Jos” University of Galati, RO-800008 Galati, Romania; ancuta.dinu@ugal.ro

**Keywords:** sensor, biosensor, polymer conductor, molecularly imprinted polymer, amino acid, tyrosine, tryptophan, phenylalanine

## Abstract

Recently, the studies on developing sensors and biosensors—with an obvious interdisciplinary character—have drawn the attention of many researchers specializing in various fundamental, but also complex domains such as chemistry, biochemistry, physics, biophysics, biology, bio-pharma-medicine, and bioengineering. Along these lines, the present paper is structured into three parts, and is aimed at synthesizing the most relevant studies on the construction and functioning of versatile devices, of electrochemical sensors and biosensors, respectively. The first part presents examples of the most representative scientific research focusing on the role and the importance of the phenylalanine, tyrosine, and tryptophan amino acids, selected depending on their chemical structure and their impact on the central nervous system. The second part is dedicated to presenting and exemplifying conductor polymers and molecularly imprinted polymers used as sensitive materials in achieving electrochemical sensors and biosensors. The last part of the review analyzes the sensors and biosensors developed so far to detect amino acids with the aid of conductor polymers and molecularly imprinted polymers from the point of view of the performances obtained, with emphasis on the detection methods, on the electrochemical reactions that take place upon detection, and on the electroanalytical performances. The present study was carried out with a view to highlighting, for the benefit of specialists in medicine and pharmacy, the possibility of achieving and purchasing efficient devices that might be used in the quality control of medicines, as well as in studying and monitoring diseases associated with these amino acids.

## 1. Introduction

Prevention of various hereditary metabolic diseases, such as phenylketonuria (PKU), alkaptonuria, Parkinson’s disease, and orientation toward a ‘bio’ diet and a healthy lifestyle—removing the factors that lead to numerous disorders and forms of depression—represent the reasons why the present study was conducted. Amino acids (AAs), responsible for the equilibrium of the nervous system—especially phenylalanine (Phe), tyrosine (Tyr), and tryptophan (Trypt)—were analyzed with a view to detecting their lack or excess and to treating them accordingly, in due time.

Over the years, many scientific researchers have developed numerous methods through which these AAs can be detected rapidly and precisely, both in biological and in pharmaceutical products. From among these methods, mention must be made of the classical high-performance liquid chromatography (HPLC) [1,2,3,4,5], mass spectrometry [6,7,8,9], fluorimetry [10], colorimetry [7,11,12], chemiluminescence [13,14,15], Raman spectroscopy [16,17], UV-Vis spectroscopy [18], capillary electrophoresis [19,20,21], and atomic force spectroscopy [22]. Moreover, versatile methods for detecting AAs have been developed and used: electrochemical ones based on sensors and biosensors, which use cyclic voltammetry (CV) [23,24,25,26,27,28] as a detection method, chronoamperometry (CA) [29], differential pulse voltammetry (DPV) [30,31,32], square wave voltammetry (SWV) [33,34,35], and linear sweep voltammetry (LSV) [36,37].

Modern challenges for scientific researchers, both in chemistry and in pharma-medicine, consist of designing sensors and biosensors with the aid of new polymers, since the latter can contribute to determining the quality of pharmaceutical products, especially during a pandemic, when new products (with various combinations of active substances) were introduced to the pharmaceutical market to address the new, severely acute coronavirus (SARS-CoV-2) [38]. Sensors and biosensors can contribute to detecting interest analytes like AAs very rapidly and exactly [24,39]. On the other hand, the selection of materials for the construction of sensors and biosensors is of crucial importance because it can lead to solving problems such as the rapid fouling of electrodes, the overlapping of the analytes redox potentials, etc. [40]. In the last five years, numerous research groups have made major contributions to the field of electroanalysis, as well as to the field of materials science, obtaining new classes of materials, such as novel polymers, which have allowed the possibility of a wide range of analytes detection [41]. The unique physical and chemical properties of CPs and MIPs, such as versatility, adaptability, sensitivity, and adjustable architecture, have led many researchers, including our group, to apply and use these new materials to develop novel chemically modified sensors and biosensors [42]. The polymers that were used in sensors were conductor polymers (CPs)—polypyrrole (PPy) [27,28,43,44], poly(3,4-ethylenedioxythiophene) (PEDOT) [45,46], polyalanine (PANI) [47], and polythiophene (PT)—and molecularly imprinted polymers (MIPs) [48,49].

Furthermore, ample studies, as well as reviews, were carried out on detection methods used to determine AAs [15,50,51,52]. The novelty of this review resides in synthesizing the studies carried out so far regarding the detection of the three AAs in human fluids, foods, and medicines, depending on the polymer used to produce sensors and biosensors, especially focusing on CPs and MIPs since they have demonstrated notable results, and since the synthesizing method is easy, having exceptional electric, thermic, and morphologic properties.

## 2. Phe-Tyr-Trypt. Properties and Importance for the Human Body

Out of the 11 amino acids essential for the human body and the nine amino acids non-essential for the human body, only three have been subjected to the present study—Phe, Trypt (essential amino acids), and Tyr (non-essential amino acid)—because of their structural similarities and the role these AAs play for the human body. Compared with other amino acids, Phe, Tyr, and Trypt are of particular importance to the central nervous system [53]. Their use as food supplements contributes to the treatment of neurovegetative disorders, disorders that can affect a number of cognitive functions, such as memory, learning, thinking, etc. [52]. These AAs are also essential components in the production of several bioactive compounds called neurotransmitters that act on the brain [6].Thus, Phe is converted into Tyr in the human body, being substances with a hydrophobic group used in treating genetic disorders, PKU and in the biosynthesis of the main neurotransmitters (dopamine, epinephrine, and norepinephrine), while Trypt is the precursor of another important neurotransmitter (serotonin) responsible for treating insomnia and anxiety (as shown in Table 1).

One of the diseases frequently appearing in the population is depression; in this regard, a useful method could be the monitoring of the Phe, Tyr, and Trypt AAs, respectively [38]. This psychological affliction can manifest itself through various symptoms, such as concentration problems, insomnia, and sadness [57,58]. The causes of its emergence can reside in various sources: biological, genetic, environmental, and social-psychological factors [59]. Depending on the type of symptom and the nature of the cause, there are many types of depression, and each needs adequate treatment. To prevent and treat mild forms of depression (postpartum, seasonal, and premenstrual), the pharmaceutical market has developed a variety of medicinal supplements that contain the AAs focused on here—in various concentrations, as shown in Table 2.

**Figure 1:**
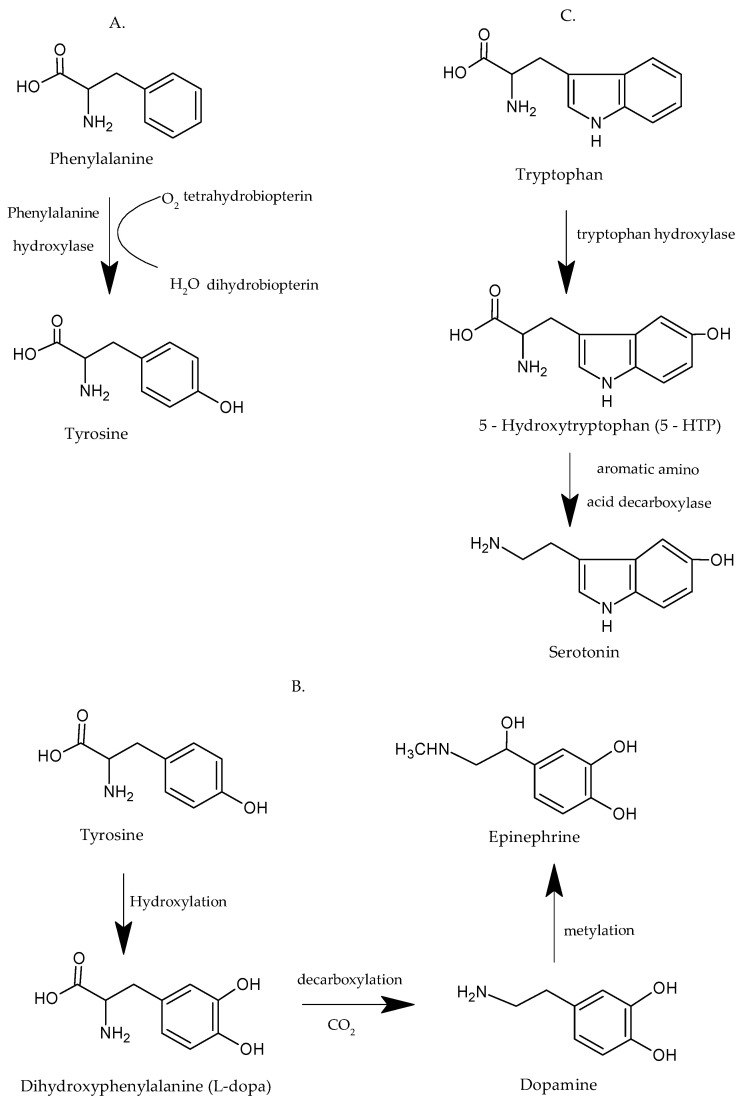
Biosynthesis of amino acids in the human body: (**A**) Phe adapted from [62]; (**B**) Tyr adapted from [67]; and (**C**) Trypt adapted from [68].

Phe, or (S)-2-Amino-3-phenylpropanoic acid, an essential AA and precursor of Tyr (Figure 1A), is assimilated by the human body through consuming foods like eggs, meat, fish, and milk, or through the administration of medicinal supplements in view of preventing Parkinson’s disease, depression, vitiligo, and attention deficit hyperactivity disorder (ADHD) [60,61,62]. Special attention should be paid to people who suffer from PKU, which is an inherited disorder caused by excessive accumulation of Phe in the human body [63]. Consequently, these people should avoid consumption of foods or supplements that contain the Phe AA, or they risk developing other disorders or diseases such as mental retardation, high blood pressure, or cerebrovascular accidents [64]. Today, there is a test for the detection of Phe, starting from birth, with sanguine serum: the Guthrie Test for the neonatal detection of PKU. It was created in 1963 by Robert Guthrie [65]. L-Phe, D-Phe, and DL-Phe are the three forms of this AA, namely the natural form, the synthetic form, and the form found in pharmaceutical products, respectively [66].

Tyr, or L-2-Amino-3-(4-hydroxyphenyl) propanoic acid, a non-essential AA by comparison with Phe and Trypt, is produced naturally in the human body, even from Phe, and through hydroxylation becomes the precursor of two important neurotransmitters of the central nervous system (SNC): adrenaline and noradrenaline—as shown in Figure 1B [67]. As in the case of the other AAs, the absence of Tyr in the human body can be compensated for by consuming various foods (nuts, oat, beans, meat, fish, and wheat) or pharmaceutical products—supplements that have the role of treating PKU and neurological disorders like depression, ADHD, Alzheimer’s disease, and mental retardation [61,69,70]. Tyrosinemia and phenylketonuria are diseases that can occur as a result of excess accumulation or an insufficient amount of Tyr in the body [63]. Thus, tyrosinemia is characterized by an abnormally high level in the blood or urine of Tyr. Phenylketonuria is a condition that prevents tyrosine biosynthesis, in the sense that individuals who suffer from this condition cannot properly process Phe AA, as a result of which they cannot obtain the proper amount of Tyr [7].

Trypt, or 2-amino-3-(1H-indol-3-yl) propionic acid, is also an essential AA that the human body uses to synthesize proteins; its intake is from external sources such as foods and pharmaceutical products. It has two important functions in the human body: on the one hand, it contributes to the biosynthesis of serotonin (Figure 1C), and on the other hand, it is involved in the biosynthesis of melatonin [68,71]. The values of Trypt sanguine concentration in the human body are situated within the following normal limits: between 10 and 40 millimoles/L—that is, between 2.05 and 5.15 mg/L [72]. In the case of values under the normal limit of Trypt, various forms of depression and insomnia are triggered, and in the case of values above the normal limit of Trypt, SNC disorders appear: manic-depressive psychosis with delirium, and schizophrenia [73,74].

Another aspect shared by the three AAs is the domain they are used in. Table 3 presents, for each domain, the uses of each AA studied here. The specialized literature mentions numerous studies focusing on the three AAs. For example, in 2020, Mahmoud Alagawany et al. published a review of the nutritional significance of AAs for raising birds and keeping them healthy, representing an alternative to therapy using antibiotics [75].

Another study on AAs (with special focus on Tyr) was carried out in 2021 by Félix Javier Jiménez-Jiménez et al. They outlined a meta-analysis of methods for determining the AAs involved in Parkinson’s disease, both in the sanguine serum and in the cerebrospinal fluid. In their conclusions, they mention that high concentrations of Tyr were found in the cerebrospinal liquid, and low concentrations of Tyr were found in the sanguine plasma [87]. Moreover, in 2020, Xiaoyang Jing et al. presented methods of AA codification. The authors mentioned five categories of methods: binary codification, codification of physical-chemical properties, codification based on evolution, codification based on structures, and codification of automatic learning. They concluded that, out of the five, codification based on evolution could obtain the best results [53]. The paper, signed by Paolo Tessari et al., presented the recommended daily doses of AAs, considering that they are the main regulators in the nutrition of an adult, being present in a wide variety of foods. The conclusion of this study highlighted the benefits of vegetable food product consumption, since necessary and important quantities of AAs are found in such products (as shown in Table 4 and Table 5) [88].

In 2020, Fieke Terstappen et al. published a paper of interest in regards to studies evaluating whether prenatal supplementation with AAs can represent a promising method of growing a healthy fetus; it included studies on 22 people and 89 animals. In the authors’ reevaluation, analyses were centralized to identify oral supplementation with AAs, the most efficient from the standpoint of the dose administered being highlighted. It was therefore concluded that the AAs in the arginine family, or BCAA (branched chain AAs), normalize the underdeveloped fetus, while the methyl-donating AAs normalize the excessive growth of the fetus [89].

In conclusion, AAs gained the title of the most important nutrients for the human body, representing “the elements which form our life” and offering the human body, alongside vitamins and minerals, the material needed to repair muscles, organs, or any other of its tissues.

## 3. CPs and MIPs Used to Determine Phe, Tyr, and Trypt

**Figure 2:**
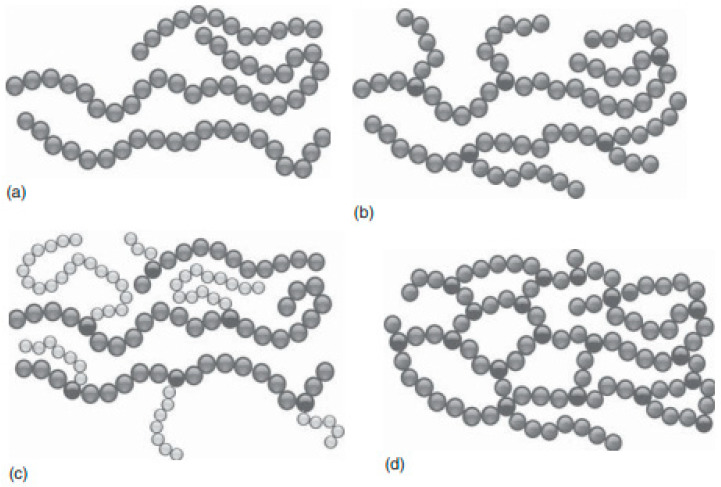
Types of polymers with various strand topologies: (**a**) linear polymer, (**b**) branched polymer, (**c**) graft polymer, and (**d**) reticulated polymer. Reprinted with permission from [91].

Known as macromolecular compounds, polymers may be found in almost all the materials that people use in everyday life. In essence, polymers are made up of several small molecules—called monomers—linked to form long strands [41]. Since they are applied in numerous fields (science, industry, and technology), the importance of polymers has been emphasized in many published articles, the advantages of their use residing in thermal stability, processability, various optic and mechanic properties, and relatively inexpensive and easy manufacturing [40]. Naturally, therefore, these versatile compounds have been used to increase the rate, stability, and sensitivity of various devices with applicability in biomedicine and bioengineering [90]. Furthermore, more types of polymers have been identified in keeping with their chemical structure, molecular mass, origin, and strand topology (as shown in Figure 2) [91].

The new areas in which polymers play a significant role are represented by biochemistry, pharmacy, biomedicine, molecular biology, and biophysics [40]. For example, in the pharmaceutical field, a polymer could be used to precisely release the active substance in a medicine [92]. In recent years, polymers have been studied in fields of research that involve the manufacturing of sensors and biosensors, endowing them with properties such as increased conductivity, and improved kinetics of electron transfer and of electrocatalytic activity [93,94]. This is the reason why the present study highlights the importance of CPs and MIPs in achieving various versatile devices for the quantification of AAs in pharmaceutical products, foods, and biological fluids.

**Figure 3:**
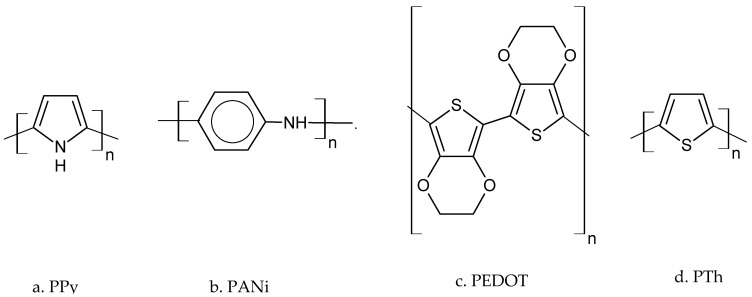
The most frequently used conductor polymers: (**a**) PPy, (**b**) PANI, (**c**) PEDOT, and (**d**) PTs. Adapted from [95].

Thus, CPs, also known as “synthetic metals”, represent a new generation of polymers, electrochemical synthesis being the preferred method of obtaining them since it has the advantage of simplicity and the possibility of achieving polymeric films of various thicknesses and doping levels [95]. Following the discovery of CPs, Alan J. Heeger, Alan G. MacDiarmid, and Hideki Shirakawa received the Nobel Prize for Chemistry in 2000 [96]. The CPs most frequently encountered in scientific research are PPy [97,98,99,100,101], PANI [102,103,104,105], PEDOT [46,106,107,108,109], PT [110,111,112,113], and polyacetylene [114,115,116], the chemical structures of which are shown in Figure 3. This type of polymer is usually obtained by electrochemical polymerization, a process that takes place in a solution that includes the solvent, the polymerizable monomer, and the electrolyte. Electropolymerization can be performed through either potentiostatic, galvanostatic, or multi-sweep techniques [117].

**Figure 4:**
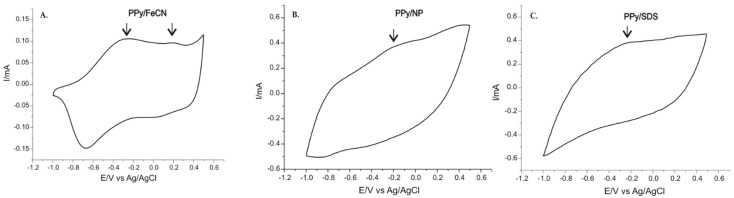
Responses of sensors modified with PPy immersed in a 0.1 M KCl and 10^−3^ M L-Tyr la 0.1 V × s^−1^ solution: (**A**) PPy/FeCN-SPCE, (**B**) PPy/NP-SPCE, and (**C**) PPy/SDS-SPCE [27].

This category of polymers has drawn the attention of many researchers, particularly because of their main property: electrical conductivity. This property of polymers is based on the presence of conjugated double bonds between carbon atoms along the polymer chain, and this bond can alternatively be single and double. Thus, a process of doping the polymer creates the conducting properties of the electrical charge [96]. Along these lines, the authors of the present paper carried out a study, published this year, that presents the manufacturing of a sensor to detect the L-Tyr AA in pharmaceutical products with the aid of the PPy conductor polymer and three doping agents: potassium hexacyanoferrate (II) (FeCN), sodium nitroprusside (NP), and sodium dodecyl sulphate (SDS). Two methods were used: chronoamperometry for the deposit on electrodes of the polymer doped with various anionic agents, and cyclical voltammetry for the electrochemical characterization of the sensors achieved. The devices obtained demonstrated good sensitivity and selectivity in detecting L-Tyr, having the following detection limits: 8.2 × 10^−8^ M for PPy/FeCN-SPCE, 4.3 × 10^−7^ M for PPy/NP-SPCE, and 3.51 × 10^−7^ M for PPy/SDS-SPCE (as shown in Figure 4) [27].

**Figure 5:**
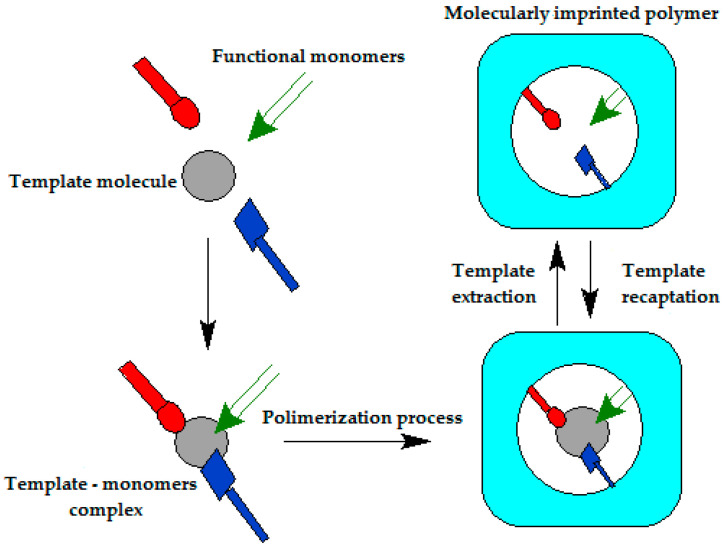
General method of preparation of MIPs. Adapted from [118].

**Figure 6:**
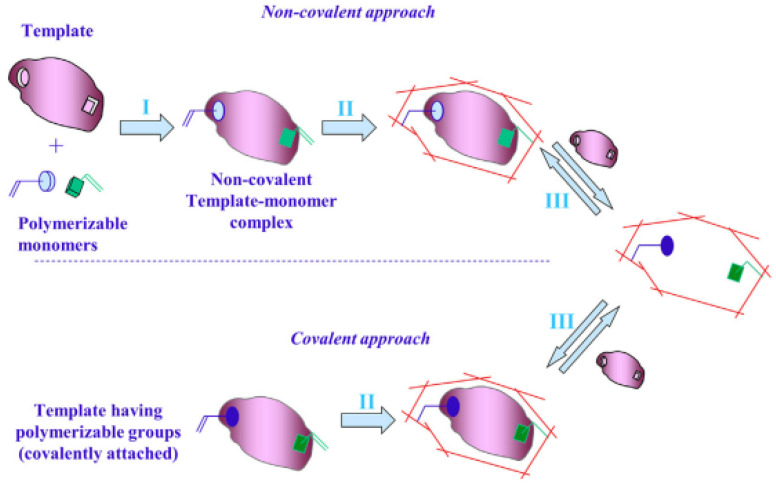
The general scheme for obtaining an MIP. Reprinted with permission from [120].

On the other hand, other polymers involved in numerous studies are MIPs in monomer solutions with template molecules, reticulation agents, or solvents, this being a versatile preparation method that can frequently be used to configure various biomimetic receivers (as shown in Figure 5 and Figure 6) [43,118]. Initially, these MIPs were synthesized by thermal heating, but because of the disadvantages of the long synthesis time and excess internal energy of the system, other methods for the synthesis of MIPs were developed, such as photopolymerization, electropolymerization, ultrasound-assisted synthesis, and microwave-assisted synthesis [119].

**Figure 7:**
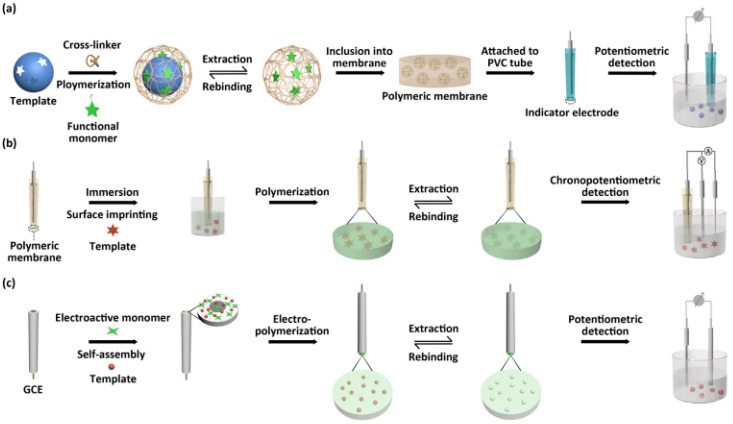
Typical manufacturing of MIP potentiometric sensors with polymeric membranes, through three processes: MIP incorporation (**a**), MIP covering (**b**), and MIP electropolymerized (**c**). Reprinted with permission from [42].

Generally, MIPs are stable, and resistant to various pH values and temperatures, but are also in various solvents [96]. Another advantage of MIPs is their relatively simple and inexpensive synthesis, which represents an alternative in using natural biological receivers [121]. Due to their affinity and selectivity, MIPs have proved to be adequate receivers for various organic and biological species such as enzymes and antibodies, and, in recent years, they have been used to manufacture electrochemical sensors and biosensors, a model for preparation being given in Figure 7 [42].

**Figure 8:**
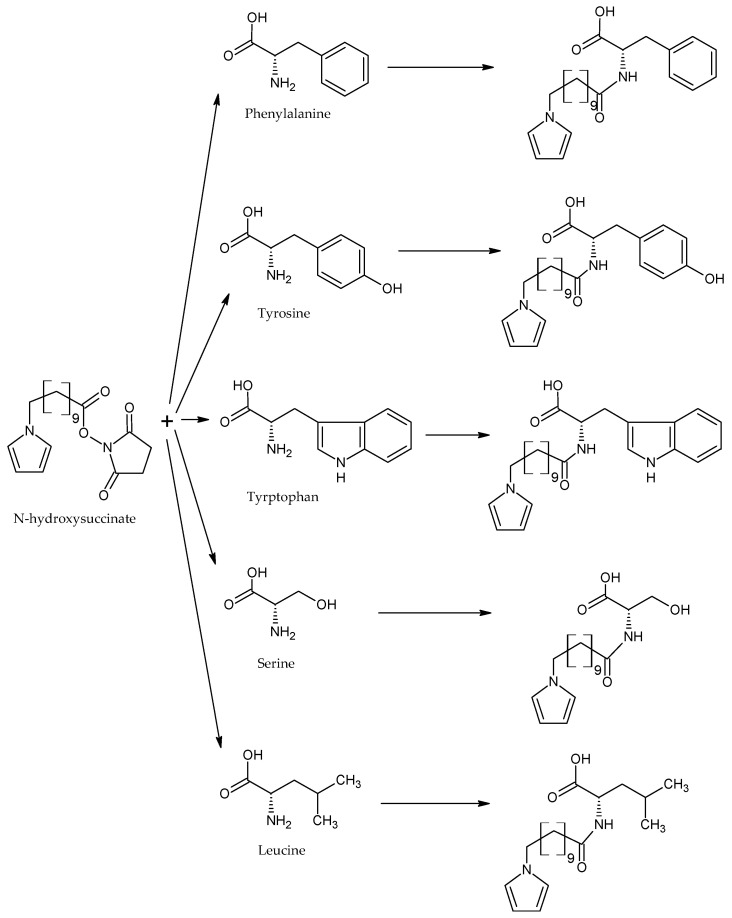
Reactions between N-hydroxy succinate and AAs. Adapted from [50].

Regarding the AAs tackled in this present study, A. Nan et al. reported (in a paper published in 2000) the synthesis and characterization of hybrid magnetic nanostructures for the analysis of AAs: Phe, Tyr, Trypt, leucine, and serine—used to functionalize the pyrrole monomer, being linked through various types of hydrophobic linkers in the azoth atom of the pyrrole monomer [50]. The methods for the characterization of these nanostructures were FTIR spectroscopy, transmission electronic microscopy (TEM), and magnetic measurements. N-hydroxyl succinate was the precursor used to obtain the monomers of pyrrole functionalized with AA, as shown in Figure 8.

**Figure 9:**
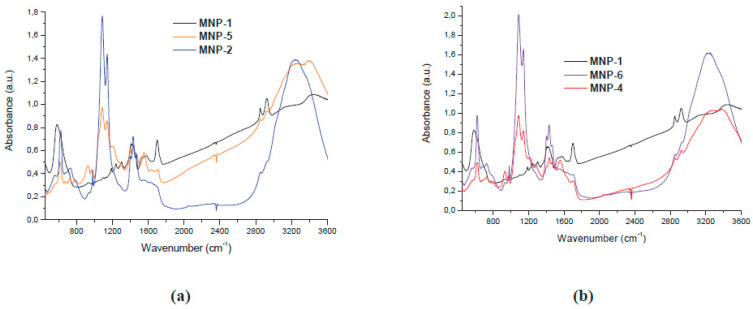
FTIR spectra of (**a**) tryptophan functionalized MNP-2, serine functionalized MNP-5, and (**b**) phenylalanine MNP-4 and tyrosine MNP-6. Reprinted with permission from [50].

**Figure 10:**
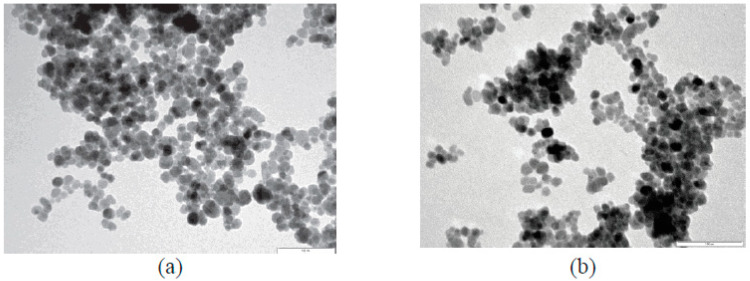
TEM image for (**a**) MNP-1 (bar-size 100 nm) and (**b**) magnetic core-shell nanoparticles based on PPy functionalized with MNP-2. Reprinted with permission from [50].

Three stages were necessary to prepare these hybrid nanoparticles based on functionalized PPy: magnetic nanoparticle synthesis (MNS), synthesis of pyrroles functionalized with Trypt, leucine, phenylalanine, serine, and Tyr, and copolymerization of functionalized pyrroles in the presence of magnetite MNP [50]. Figure 9 shows the results obtained through FTIR in the process of preparing hybrid magnetic nanoparticles (MNP) based on functionalized polypyrrole. The results obtained demonstrate a high level of magnetic nanoparticle dispersibility, a uniform dimension, and a spherical shape, as shown in Figure 10. In conclusion, the authors proved the superparamagnetic behavior for the functionalized magnetic nanostructures based on functionalized PPy.

Since the three AAs are found in biological fluids, implicitly in human blood serum and urine, it is extremely important to monitor their levels in the body, to measure their concentration by means of more sensitive and more selective devices such as sensors and biosensors.

## 4. Sensors and Biosensors Based on CPs and MIPs to Quantitatively Determine AAs (Phe, Tyr, and Trypt)

### 4.1. General Methods Used to Determine AAs

Many scientific articles, reviews, book chapters, and volumes about how to detect Phe, Tyr, and Trypt have been published so far. Each scientific paper describes unique methods of AA detection, which, as technology has advanced, highlighted advantages and disadvantages. In compiling the data in Table 6, a series of method performance criteria were in view: precision, selectivity, accuracy, sensitivity, detection limit, cost, and duration, classified according to the intensity of each method.

The disadvantages connected with electrochemical methods have stimulated researchers to improve the properties and performances of sensors and biosensors using, for the quantitative determination of AAs, Phe, Tyr, and Trypt, resorting to their modification, either with CPs doped with electroactive ions or with MIPs. Thus, researchers highlighted the unique properties of these devices: their optical, electrical, and mechanical properties, increased stability, high response rate, and increased sensitivity in the process of rapidly and precisely detecting AAs [127]. These analytical instruments were therefore found applicable in a large range of fields, including biotechnology and bio-pharma-medicine [93,94,121].

In short, the specific goal of this review resided in synthesizing the articles published so far in which CPs and MIPs were involved to develop electrochemical sensors and biosensors used to detect the three amino acids mentioned: Phe, Tyr, and Trypt.

### 4.2. CPs and MIPs Involved in Developing Electrochemical Sensors to Detect AAs: Phe, Tyr, and Trypt

**Figure 11:**
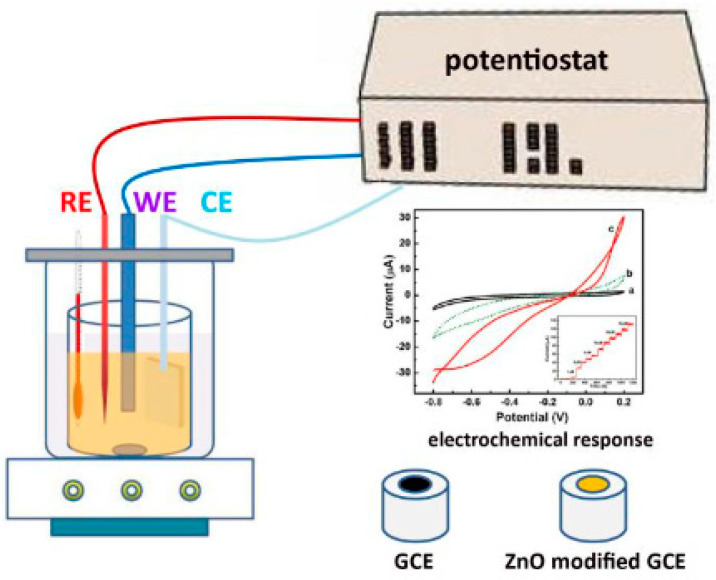
Schematic illustration of the equipment used for analysis with an electrochemical sensor. Reprinted with permission from [130].

Sensors and biosensors, high-interest instruments, are used in many research fields: medicine, pharmacy, industry, transport, environmental protection, and automation. Thus, in the future humanity will depend on many of these devices (with people who suffer from diabetes depending on glucometers—devices that detect the glycaemia levels in the body—constructed with the aid of a biosensor) [103,128]. Thus, the stage of selecting sensor construction/manufacturing materials is extremely important, as the materials can contribute to solving various problems related to analyte detection, such as the redox potential of molecules, the deterioration of electrode surfaces—leading to low reproducibility. To improve various properties—such as electrical conductivity, mechanical stability, and chemical surface—electrochemical sensors were achieved with the aid of a wide range of materials like CPs, applying the following electrochemical methods: potentiometry [66], conductometry [94], amperometry [103], and voltammetry [36,129]. This category of sensors is used especially in systems for monitoring the environment and health, in food quality control, and in the general scheme of the equipment used to electrochemically analyze an electrode, as illustrated in Figure 11 [130].

In 1959, chemist Jaroslav Heyrovsky received the Nobel prize for discovering the polarographic voltammetric method, which allowed the further development of other electroanalytical techniques such as CV, DPV, LSV, and SWV [129]. These methods showed a series of advantages through the years: simultaneous determination of more analytes, increased sensitivity with regard to detecting organic and inorganic species in various concentration ranges, the ability to work with a large range of temperatures, the capacity to determine kinetic parameters and to estimate unknown parameters, and rapid analysis [131,132]. Due to these advantages, this review has summarised, in Table 7, the notable studies developing sensors characterized through voltammetric methods and constructed based on CPs and MIPs to detect the three AAs.

According to Table 6, Funda Alışık et al. contributed to detecting AA L-Phe by preparing polyurethane sensors based on Arabic gum, modifying platinum electrodes using the electropolymerization technique. It was analyzed through DPV, showing increased sensitivity and reproducibility in detecting a wide range of L-Phe concentrations. The development of such a sensor was considered useful for selectively detecting PKU, the sensor being analyzed and validated by numerous techniques, such as FTIR, DTA, TGA, and SEM. The novelty of the research is represented by the polyurethane polymer, which gives the sensor good adhesion and a selective permeability. [134].

Tatiana V. Shishkanova et al. used the β-cyclodextrin pyrrole polymer in preparing the sensor used to molecularly recognize Phe enantiomers. In this case, the electrochemical method used was LSV, in the 0.1–0.75 × 10^−6^ M (*n* = 3) concentration range, manifesting higher sensitivity for the D-Phe enantiomer as compared to the L-Phe one. This study was based on the characterization, deposition, and recognition of the properties of the modified CP (pyrrole-β-cyclodextrin conjugate)-modified sensor [135].

Yu-fang Hu et al. developed an electrochemical sensor in whose fabrication CP PANI was involved, the electrochemical behavior of the sensor being studied through CV and DPV methods in view of using it to determine L-Phe in human serum samples. The sensor demonstrated excellent stability, sensitivity, selectivity, recuperation, and reproducibility. The research developed a new electrochemical printing technique using a PANI-coated electrode, a stable conductive polymer with high electrocatalytic ability [102]. Other researchers developed sensors to detect L-Phe through the molecular imprinting technique. Along these lines, Funda Alışık et al. obtained, for the 20 sensors prepared, a stable reproducibility percentage of 97.67%, with an RSD value of 2.33%, thus demonstrating that the sensor prepared from p-toluene sulphonic acid (PTSA) polymeric films had high stability, repeatability, and selectivity for L-Phe [134]. Nihal Ermiş et al. used the Thiophen-3-carbonyl tryptophan (TP3C-Trp) monomer, developing electrochemical sensors characterized through CV, drawing a parallel between non-imprinted sensors (NIP) and imprinted ones (MIP) to selectively and sensitively determine L-Phe. The linearity range obtained was wide, 1.0 × 10^−8^–1.0 × 10^−7^ M, and proved to be useful in detecting L-Phe in egg whites and chicken samples. By electropolymerizing the polymer, the authors with the help of TP3C-Trp developed a new sensor for Phe detection [43]. Another sensor prepared through the molecular imprinting technique belongs to Yasuo Yoshimi and Noriyuki Ishii, who discovered enantioselective sensitivity to Phe in water solution through the cyclic voltametric method, at the same time using a mixture of reticular hydrophobic (hydrophobic ethyleneglycol dimethacrylate) and hydrophilic (hydrophilic methylene bisacrylamide) agents, which demonstrated improvement of sensor sensitivity. The concentration range used was 3–5 × 10^−6^ M, demonstrating the utility of MIP for molecular recognition in biomimetic sensors. The development of such an anilide-printed poly (ethylene glycol dimethacrylate (EDMA) co-methacrylic acid (MAA))-based electrode was intended to demonstrate the possibility of chiral-selective detection of Phe using MIPs, using a crosslinked monomer combination [136].

To determine Tyr, the CPs most frequently involved in developing electrochemical sensors were PEDOT and PPy. Thus, F. Nada et al. carried out a study in which sensors were tested on real samples of biological fluids to determine four analytes: norepinephrine, paracetamol, Tyr, and ascorbic acid. Each component that contributed to sensor preparation had unique characteristics that conferred the devices a remarkable electro-catalytic activity. CP PEDOT was used to endow the sensor with increased electrical conductivity and stability [45]. The linear range obtained for detecting Tyr was 0.06–20 × 10^−6^, and the detection limit was low; it was therefore considered that this device would be attractive and useful in the medical field. The novelty of the study is underlined by the advantages of the PEDOT, multi-walled carbon nanotubes, Nafion, and crown that it gives to the new sensor prepared by electrochemical polymerization. In the papers published by Nathiya Dhananjayan et al. (2019) [139] and Ramya, R. et al. (2018) [137], PPy was used as CP in fabricating sensors for the ultrasensitive detection of Tyr. Both articles demonstrated that the PPy polymer contributed considerably to improving the properties of sensors through increased stability, conductivity, sensitivity, and better biocompatibility. In the first case, the sensor was applied to determine the concentration of Tyr in human urine samples, chicken meat, and cow’s milk; in the second case, it was applied to tea and chicken meat samples. In a study by Nathiya Dhananjayan et al., a sensor was developed based on a biopolymer, namely modified gum acacia, encapsulated with electron-beam-irradiated polypyrrole nanospheres, and in the Ramya study, R. et al. applied a new synthesis of electron-beam-irradiated polypyrrole modified with sheets over bovine serum albumin. [137,139].

Furthermore, sensors prepared through the molecular imprinting technique are present in this case also. For example, Bangjie Chen et al., obtained a linearity range of 1 × 10^−8^ M to 4 × 10^−6^ M for determining Tyr. A carbon electrode was molecularly imprinted with a polyaniline/polythionine/gold nanoparticle@zeolitic imidazolate framework-67 composite, and analyzed with a cyclical voltametric method on human serum samples, with satisfactory results: 98.8%. The materials used in the molecular imprinting were selected because of their large surfaces, high porosity, and biocompatibility [47]. In the case of the study carried out by Nihal Ermiş et al., the molecular imprinting was achieved with PPy films on a gold electrode, with excellent results obtained on the human plasma samples used to detect Tyr, demonstrating good reproducibility and repeatability [140]. On human urine samples, Varghese Saumya et al. applied the MIPPy/GCE sensor, prepared and analyzed on site through an electrochemical method, which had the advantage of increased simplicity and sensitivity. The concentration range used for Tyr was 1 × 10^−8^ to 8 × 10^−6^ M, and the sensor was applied to detect tyrosine in human urine samples [138].

**Figure 12:**
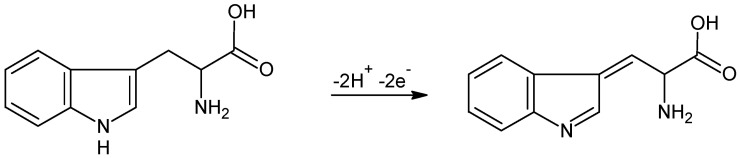
Process of the electrochemical oxidation of L-TRP by PPy/FeCN/SPCE sensor [28].

To identify and quantify tryptophan with sensors based on conductor polymers and molecularly imprinted polymers, a series of studies were carried out, with applicability on the following types of real samples: human urine [141], biological fluids [143,144], and pharmaceutical products [28,49]. In Figure 12 is presented the detection principle of a voltametric sensor based on polypyrrole doped with ferrocyanide ion.

The studies demonstrated the increased performance of the devices achieved, mainly due to using CP or to the diversity of the molecular imprinting materials.

All the benefits of the conducting polymers demonstrate their ability to integrate into micro/nano devices in order to detect or monitor different bioanalytes. The various types of conducting polymers make it possible to couple them with various biological and/or chemical species to obtain high performance characteristics, such as improved sensitivity and selectivity. The progress observed is closely related to the selection of the type of polymers, the processing technologies that aim to integrate CPs on the surface of (bio)sensors with wide applications in various applicative fields [93].

Thus, we found in the literature that one of the major challenges in the development of an electrochemical (bio)sensor based on CP is represented by the immobilization of the transducer on the electrode surface in order to achieve a good transduction of the signal [145]. As a result, the mechanical properties of CP films and the effects of thickness and microstructures on them, and breaking behaviour in the presence of thermal and mechanical factors, should be taken into account when a CP is selected [146].

### 4.3. CPs and MIPs Involved in Developing Electrochemical Biosensors to Detect AAs: Phe, Tyr, Trypt

As mentioned by the authors of the research described in this section, the application of CPs for the design of MIPs and the various possibilities for the immobilization of biological recognition elements, such as enzymes, antibodies, or proteins, are important advantages of biosensors based on CPs, finding applicability in many directions of research [147]. So, the challenge in the principle of the selection of conducting polymers used in the manufacture of biosensors is closely related to the method of production, the enzyme used, and the analytes to be detected [148]. The polymer matrix provides a suitable environment for the immobilization of the enzyme, while maintaining its long-term activity, especially in electrochemical measurements [149].

If the sensor is an analytical instrument that translates physical and chemical data into measurable signals, biosensors play the same role, but are based on a combination of a biological recognition compound and a physical translator—the recognition element being either an enzyme, an antibody, or a microorganism—which renders it more sensitive for detecting the substance analyzed. Immobilization methods of biomolecules include covalent binding, crosslinking, entrapment, adsorption, and affinity. All the methods have advantages and disadvantages, but one of the most important aspects to be taken into account is the maintaining of the bioactivity of the biomolecules [150].

**Figure 13:**
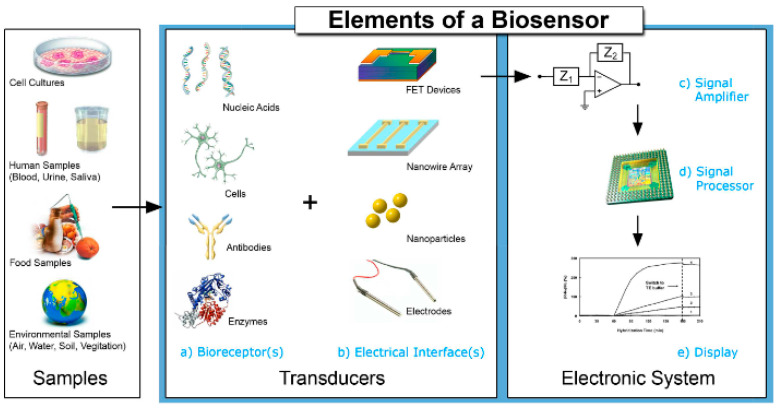
Main elements of a biosensor. Reprinted with permission from [151].

The typical scheme of a biosensor is presented in Figure 13 [151].

Therefore, a biosensor is a device designed to obtain a digital electronic signal proportional to the concentration of a chemical compound in the presence of an interfering species. Their difference from the sensors is even written with the prefix “bio”, precisely because of their biofunctionality, respectively biocatalysis and molecular recognition, and this aspect led to a typical biosensor architecture represented by two types of components: the biological component, and the transducer component [152].

Biosensors are applied to a variety of samples: biological fluids, food samples, medicine samples, cellular cultures, or environment samples [153]. Their sensitivity is higher in comparison with sensors because of the biological recognition compound [127]. In this section also, the criteria for scientific paper selection were represented by the use of CPs and MIPs, AA (Phe, Tyr, Trypt) detection, and the use of voltametric methods.

Thus, in 2018, C.S. Pundir et al. compiled a review on the determination of the D and L enantiomers of amino acids with the aid of biosensors. They mentioned the optimum functioning parameters used to detect AAs: the 5.3–9.5 pH interval, the 25–45 °C temperature interval, the 0.0008–8000 × 10^−6^ M AA concentration interval, the 0.02–1250 × 10^−6^ M detection limit, and the −0.05–0.45 V work potential between 2 s and 900 s. AAs were detected in fruit juices, beverages, urine, and blood serum, the biosensors showing a 200 times repeatability during an interval of between 7 and 120 days [127]. Moreover, Table 8 presents other studies in which biosensors with CPs and MIPs were achieved to detect AAs through voltametric methods.

**Figure 14:**
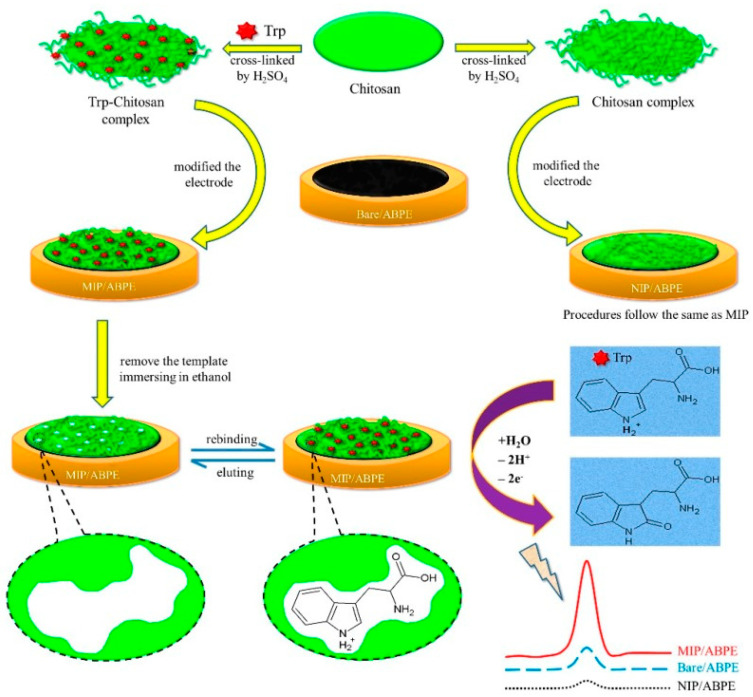
The procedure for the development of the MIP/acetylene black paste electrode and the principle of Trypt detection [159].

The detection principle of the MIP-based sensors could be mainly impedimetric, voltametric, or amperometric. In Figure 14 is presented the detection process of the Trypt with a MIP-based sensor.

Biosensor studies in which CPs and MIPs were involved, developed to determine the three AAs, are less numerous than the studies on electrochemical sensors. Thus, for the Phe AA, quartz crystal electrodes molecularly imprinted with copolymer, polyacrylonitrile, and acrylic acid were used. Their analysis was carried out in parallel with a series of non-molecularly imprinted copolymer electrodes, emphasizing higher sensitivity in the case of the poly(AN-co-AA)-modified biosensor, (0.5839 Hz/mgL^−1^), as compared to the non-imprinted one, −0.2724 Hz/mgL^−1^, and reproducibility (RSD) was 1.84%. Biosensor selectivity was demonstrated by simultaneous testing of analytes: Phe, dopamine (DA), ascorbic acid (AscA), vanillylmandelic acid (VMA), uric acid (UA), Trypt, and Tyr. This study was conducted by Ablolreza Mirmohseni et al. in 2008, stating that the developed biosensor could be successfully applied to human serum samples [155]. The novelty of the research is in the use of poly (AN-co-AA) polymer to detect the level of Phe in different solutions, compared to a study done prior to this research, in which the polymer was applied for the racemic separation of Phe [160].

A representative study for the chiral recognition of L/D-Tyr and L/D-Trypt with biosensors was signed by Lijun Zhang et al. They proposed a model of electrodes modified with MIP films and organic electrochemical transistors (OECTs). Selectivity toward the L-Trp, D-Trp, L-Tyr, and D-Tyr enantiomers was 11.6, 3.5, and 14.5, respectively, 2.6 × 10^−6^ M, the MIP films bringing a remarkable contribution to obtaining these values [156]. The study’s authors present a new approach to the quantitative recognition of Tyr and Trypt enantiomers, constructing a biosensitive chiral electrochemical system in which the synergistic and complementary effect of L-DHCNT/L-Cys and D-DHCNT/D was analyzed (left-/right-handed double helix carbon nanotubes@Polypyrrole@Au na-noparticles@L/D-Cysteine) on this system, influencing the potential and intensity of the signal. The study presents a new approach to the quantitative recognition of Tyr and Trypt enantiomers, constructing a biosensitive chiral electrochemical system in which the synergy and complementary effects of L-DHCNT/L-Cys and D-DHCNT/D-Cys were analyzed (left-/right-handed double helix carbon nanotubes @ Polypyrrole @ Au nanoparticles @ L/D-Cysteine) on this system, influencing the potential and intensity of the signal. The research carried out in view of obtaining portable, sensitive, and precise devices is in constant development and regards multiple areas of interest (medicine, pharmacy, chemistry, biochemistry, and the food industry). In connection with determining the Phe, Tyr, and Trypt AAs in various real samples (medicines, foods, and biological samples), the emphasis lies on the use of a new generation of materials such as CPs and MIPs because of their excellent properties.

As mentioned in the literature, the application of CPs for the design of MIPs and the various possibilities of immobilization of biological recognition elements, such as enzymes, antibodies or proteins, are important advantages of biosensors based on CPs, giving them applicability in many fields of research [161].

The principle for the selection of conducting polymers used in the development of biosensors is closely related to the method of fabrication, the enzyme, or other biological recognition elements used and the analytes to be detected [162]. For instance, the polymer matrix provides a suitable environment for immobilizing the enzyme, which maintains its long-term activity, especially in the electrochemical measurements [163].

## 5. Conclusions and Future Developments

This critical analysis synthesizes and describes the main sensors and biosensors achieved with the aid of various relatively new polymer classes, namely CP and MIP—which have remarkable sensitive properties: electrical conductivity, increased stability, and biocompatibility. The molecular imprinting technique is based on manufacturing synthetic receivers with the capability of recognizing a certain analyte, and with electrochemical or optical detection. CPs are mainly used to develop voltametric and potentiometric sensors. Due to the high level of interest in the field, the study concentrates especially on the detection of three AAs (Phe, Tyr, and Trypt), as humanity is inflicted with various forms of depression caused by the lack or the excess of these AAs—afflictions that are increasingly more difficult to manage. In conclusion, the sensitive and precise quantification of AAs to evaluate the quality and authenticity of pharmaceutical products, beverages, and foods, alongside their physiological and nutritional importance, has stirred interest in many researchers. Furthermore, attention was paid to developing versatile systems for analyzing and rapidly detecting AAs, and the electroanalytical methods employed demonstrated efficiency, precision, and low costs.

Future research developments are oriented toward achieving, improving, and marketing these kinds of sensitive devices—useful not only for each individual, but for the European Medicines Agency also—in controlling the quality of various products with amino acid content. In regards to the technical challenges, they are mainly related to developing functionalized polymers that have the possibility to selectively interact with the target amino acid. This new type of polymer can be useful both for molecularly imprinted polymers—polymers that represent the sensitive material—and for polymers that represent the support for biological element immobilisation, such as enzymes, nucleic acids, or antibodies. Achieving functionalized nanocomposite polymers—carbon nanomaterials—is another method that can be applied and lead to increasing the selectivity of sensitive devices.

The detection performance can also be improved by using new techniques that are more rapid and more sensitive, such as ultra-fast cyclical voltammetry, or through combining the detection techniques—as is the case with the spectroelectrochemical technique, which combines voltammetric techniques with UV-Vis or Raman spectroscopy. Accessing and applying information from various fields can prove useful in the process of detection and quantification.

## Figures and Tables

**Table 1:** Chemical structure and physical-chemical properties of AAs: Phe, Tyr, and Trypt.

Amino Acid	Chemical Structure	Chemical Formula	Chemical and Physical Properties	References
Phenylalanine	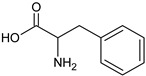	C_9_H_11_NO_2_	Aromatic Non-polar M_w_ = 165.19 g/mol Hydrophobic substance	[54]
Tyrosine	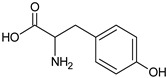	C_9_H_11_NO_3_	Aromatic Non-Polar M_w_ = 181.19 g/mol Hydrophobic substance	[55]
Tryptophan	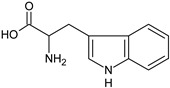	C_11_H_12_N_22_	Aromatic Non-polar M_w_ = 204.22 g/mol Hydrophobic substance	[56]

**Table 2:** Pharmaceutical products that contain amino acids Phe, Tyr, and Trypt.

Amino Acid	Pharmaceutical Products	Concentration/Capsule	Producer/Country
Phenylalanine	Amino 75	75 mg	SOLGAR/USA ^2^
	L-Phenylalanine 500	500 mg	SOLARAY/USA
	DLPA ^1^ 500	500 mg	SOLGAR/USA
	Best D-Phenylalanine	500 mg	DOCTOR’S BEST/USA
Tyrosine	L-Tyrosine 500	500 mg	SOLARAY/USA
	Tiroidin	90 mg	PARAPHARM/ROMANIA
	Cebrium	4.12 mg	NEUROPHARMA/GERMANY
	Thyroid Caps	100 mg	SOLARAY/USA
Tryptophan	Sleep Optimizer	150 mg	SOLARAY/USA
	Cebrium	0.2 mg	NEUROPHARMA/GERMANY
	L-Tryptophan	500 mg	SOLARAY/USA
	Tonico Vita	18 mg	TERAPIA/ROMANIA
	MaxiMag Women	150 mg	ZDROVIT/ROMANIA

^1^ DLPA, DL Phenylalanine; ^2^ USA, United Stated of America.

**Table 3:** The uses of AAs: Phe, Tyr, Trypt.

Domain of Use	Uses			References
	Phe	Tyr	Trypt	
Chemistry Medicinal	Depression, ADHD ^1^, Parkinson’s disease, chronic pain, osteoarthritis, rheumatoid arthritis, alcohol withdrawal symptoms, and vitiligo skin disease	Phenylketonuria mental performance, alertness or memory, depression, or ADHD	Premenstrual dysphoric disorder syndrome, sleep problems (insomnia), anxiety, depression, and ADHD	[76,77,78,79,80,81]
Pharmacology Pharmacy	Is part of medicinal supplements under various forms: capsules, creams, vials, and syrups.			[26,28,82,83,84,85,86]

^1^ ADHD, attention deficit hyperactivity disorder.

**Table 4:** Recommended daily doses for a 70 kg male, and AAs in various foods: animal source foods. Reprinted with permission from [88].

*RDA* ^1^		Egg 100 g	Milk 100 mL	Beef 100 g	Pig 100 g	Chicken 100 g	Sea Bass 100 g
	Protein content (g)	12.1	3.3	22	20.7	23.3	21.3
*Essential amino acids*							
2100	Lysine	1001	272	2002	1737	2246	2021
700	Histidine	322	93	849	647	937	552
1050	Threonine	674	164	898	919	1160	967
1050	Cysteine + Methionine	740	118	871	780	974	897
1820	Valine	896	233	1063	1243	1384	1044
1400	Isoleucine	741	192	950	1080	1153	914
2730	Leucine	748	355	1892	1624	1955	1655
1750	Phenylalanine + Tyrosine	1247	318	1677	1166	1776	1531
280	Tryptophan	228	50	246	183	273	249
12,880	Total EAAs (mg)	6597	1795	10,448	9379	11,858	9830

^1^ RDA, Recommended daily doses.

**Table 5:** Recommended daily doses for a 70 kg male, and AAs in various foods: vegetable source foods. Reprinted with permission from [88].

*RDA* ^1^		Soybeans 100 g	Beans 100 g	Peas 100 g	Wheat 100 g	Maize 100 g	Rice 100 g	Potato 100 g	Cauliflower 100 g	Quinoa 100 g
	Prot. content (g)	38.9	10.2	5.5	11	8.7	6.7	2.1	3.2	19.6
*Essential amino acids*										
2100	Lysine	3047	714	348	239	258	257	92	120	1025
700	Histidine	1170	303	85	228	251	165	28	37	478
1050	Threonine	1843	428	310	310	334	246	59	74	849
1050	Cyst + Meth	1183	238	95	454	307	257	51	63	565
1820	Valine	2176	616	226	452	472	438	99	104	961
1400	Isoleucine	2222	556	201	403	350	306	68	73	808
2730	Leucine	3689	885	342	741	1028	590	96	126	1399
1750	Phe + Tyr	3970	963	345	855	761	588	132	129	1542
280	Tryptophan	618	113	54	116	61	84	/	/	726
12,880	Total EAAs (mg)	19,918	4816	2006	3798	3822	2931	624	726	8353

^1^ RDA, Recommended daily doses.

**Table 6:** Performance criteria of the methods developed for the detection of AAs.

	Precision	Selectivity	Accuracy	Detection limit	Cost and Duration
**High**	Electrochemical methods based on achieving sensors and biosensors [15,37,49,92]				
**Medium**	Instrumental (electrical methods [122], optical methods [123], thermal methods [124], magnetic methods, and radiochemical methods [125]				
**Low**	Chemical methods (volumetry, gravimetry, precipitation methods) [126]				

**Table 7:** Performances of various electrochemicals sensors based on CPs and MIPs to detect the Phe, Tyr, and Trypt AAs. A summary.

AA ^1^	CPs ^2^			MIPs ^3^		
	Electrode Architecture	Detection Technique	LOD ^4^ (M)/Sensitivity/Linear Range	Electrode Architecture	Detection Technique	LOD (M)/Sensitivity/Linear Range
Phenylalanine	Sensor with Gum Arabic based polyurethane modification [133]	DPV ^5^	48.01 × 10^−6^/0.002 × 10^−6^/ 200–1000 × 10^−6^	Sensor with p-Toluene Sulfonic Acid Modified Pt Electrode [134]	DPV	0.59 × 10^−6^/ 0.003 × 10^−6^/ 2–2000 × 10^−6^
	PPy-β-CD/GCE (polypyrrole-β-cyclodextrin conjugate)/glassy carbon electrode [135]	CV ^6^, LSV ^7^	D-Phe (138 ± 15) × 10^−3^ and for L-Phe (6 ± 1) × 10^−3^/ 0.1–0.75 ×10^−3^	MIP/TP3C-Trp (molecularly imprinted polymer/Thiophen-3- carbonyl tryptophan) [43]	SWV ^8^, CV	1.0 × 10^−9^/ 2.7532 × 10^−9^/ 1.0 × 10^−8^–1.0 × 10^−7^
	β-CD–MWNTs/PAN/CE (polyaniline modified carbon electrode based on cyclodextrin incorporated carbon nanotube composite material and imprinted sol–gel film) [102]	CV, DPV	1.0 × 10^−9^/56.283 × 10^−9^/ 5.0 × 10^−7^–1.0 × 10^−4^	MIP-grafted ITO/EDMA/MBAA (electrode grafted with a molecularly imprinted polymer crosslinked via a combination of hydrophobic ethyleneglycol dimethacrylate and hydrophilic methylene bisacrylamide) [136]	CV	0.5 × 10^−6^/ 3–5 × 10^−3^
Tyrosine	GC/CNT/PEDOT/NF/Crown (glassy carbon/multi-walled carbon nanotubes/poly (3-4-ethylene dioxythiophene/Nafion/Crown) [45]	CV	0.429 × 10^−9^/ 963.1 × 10^−9^/ 0.06–20 × 10^−9^	MIP/pTH/Au@ZIF-67 (molecularly imprinted polyaniline/ polythionine/gold nanoparticles@zeolitic imidazolate framework-67 composite) [47]	DPV	7.9 × 10^−^^10^/ 0.0005 × 10^−^^10^/ 1 × 10^−8^–4 × 10^−6^
	EB-Ppy-BSA/GCE (Electron beam irradiated polypyrrole nanospheres embedded over bovine serum albumin) [137]	SWV	8.8 × 10^−9^/ 1.04 × 10^−9^/ 100 × 10^−9^–800 × 10^−6^	In situ copper oxide modified MIPPy (molecularly imprinted polypyrrole) coated GCE (glassy carbon electrode) [138]	LSV	4.0 × 10^−^^9^/ 0.47 × 10^−^^9^/ 1 × 10^−8^–1 × 10^−6^ and 2 × 10^−6^–8 × 10^−6^
	EB-PPy/MGA (Electron Beam-polypyrrole/Modified Gum Acacia) [139]	CV, SWV	85 × 10^−9^/ 18.944 × 10^−9^/ 0.4–600 × 10^−6^	MIP-PPy/AuE (molecularly imprinted polymer-polypyrrole/gold electrode) [140]	CV, SWV	2.5 × 10^−9^/ 0.6567 × 10^−9^/ 5.0 × 10^−9^–2.5 × 10^−8^
Tryptophan	CuNPs/p-TAOX/GCE (copper nanoparticles/poly(3-amino-5-mercapto-1,2,4-triazole)/glassy carbon electrode) [141]	DPV, CV	0.16 × 10^−6^/ 8.2058 × 10^−6^/ 4.0–144.0 × 10^−6^	MIOPPy/pABSA/GCE (molecularly imprinted overoxidized Polypyrrole (OPPy)/Poly (p-aminobenzene sulfonic acid) modified glassy carbon electrode [142]	CV	1.2–4 × 10^−6^
	3DCu(x)O-ZnO NPs/PPy/RGO A three-dimensional porous nanocomposite of reduced graphene oxide decorated with polypyrrole nanofibers and zinc oxide-copper oxide p-n junction heterostructures [143]	DPV, CV	0.016 × 10^−6^/ 0.1345 × 10^−6^/ 0.053–480 × 10^−6^	Nafion-MIP-MWCNTs@IL/GCE (Nafion-molecularly imprinted copolymer-ionic liquid (i.e., 1-butyl-3-methylimidazolium hexafluorophosphate) functionalized multi-walled carbon nanotubes/glassy carbon electrode) [49]	DPV, LSV	6 × 10^−9^/ 5.09 × 10^−9^/ 8 × 10^−9^–26 × 10^−6^
	PPy/FeCN/SPCE (polypyrrole/potassium hexacyanoferrate (II))/carbon screen-printed electrode) [28]	CV	1.05 × 10^−^^7^/ 0.87268 × 10^−^^7^/ 3.3 × 10^−7^–1.06 × 10^−5^	MIP -MWCNT s/GCE (molecularly imprinted polymer-modified modified with multi -walled carbon nanotubes/glassy carbon electrode) [144]	CV	1.0 × 10^−9^/35.863 2 × 10^−6^, 1.114 2 × 10^−6^, 0.1635 2 × 10^−6/^ 2.0 × 10^−9^−0.2 × 10^−6^, 0.2 × 10^−6^−10 × 10^−6^ and 10 × 10^−6^−100 × 10^−6^

^1^ AA, amino acid; ^2^ CPs, conductive polymers; ^3^ MIPs, molecularly imprinted polymers; ^4^ LOD, limit of detection; ^5^ DPV, differential puls voltammetry; ^6^ CV, cyclic voltammetry; ^7^ LSV, linear sweet voltammetry; ^8^ SWV, square wave voltammetry.

**Table 8:** Performances of biosensors with CPs and MIPs to detect Phe, Tyr, and Trypt.

AA	CPs			MIPs		
	Electrode Architecture	Detection Technique	LOD (M)/Sensitivity/Linear Range	Electrode Architecture	Detection Technique	LOD (M)/Sensitivity/Linear Range
Phenylalanine	L-AAOD-polytyramine electrode (L-amino acid oxidase) [154]	CV	0.07 × 10^−6^/ 0.07–3 × 10^−3^	MIP/acid (poly(AN-co-AA)/QCN electrode (quartz crystal nanobalance electrode imprinted polyacrylonitrile and acrylic) [155]		45 mgL^−1^/ 0.5839 Hz/mgL^−1^/ 50~500 mgL^−1^
				L-Phe-IPDA-CdS-CdSe-Zn/Ti PEC (L-Phe-imprinted polydopamine-coated Zn/CdS/CdSe/heterojunction) [147]	CV, CA ^1^	0.9 × 10^−9^/ 0.005–2.5 and 2.5–130 × 10^−6^
Tyrosine	Polythreonine-modified graphite-carbon nanotube paste electrode [148]	CV, DPV	2.9 × 10^−7^/ 9.92 × 10^−7^ 2 × 10^–6^ to 2.5 × 10^–5^ and 3 × 10^–5^ to 1.2 × 10^–4^	MIP-OECTs (molecularly imprinted polymer-organic electrochemical transistors) [149]	CV	30 × 10^−9^/ 14.5 and 12.5/ 300 × 10^−9^ to 10 × 10^−6^
	L/D-DHCNT@PPy@AuNPs@L/D-Cys (left-/right-handed double helix carbon nanotubes/Polypyrrole@Au nanoparticles nanocomposites/L/D-cysteine) [156]	DPV	1.88 × 10^−1^ L-Tyr and 5.72 × 10^−1^ D-Tyr/−0.004			
	D-CNT@PPy@Pt NPs@beta-CD (polypyrrole-coated chiral carbon nanotubes with Pt nanoparticles and beta-cyclodextrin) [157]	CV	0.107 × 10^−9^/ 3–30 × 10^−6^			
Tryptophan	PT-Ag/L-Try/GCE (polythiophene with silver dendrites composite/L-Tryptophan/glassy carbon electrode) [110]	CV, SWV	20 × 10^−9^/ 200 × 10^−9^–400 × 10^−3^	MIP-QCM biosensor (molecularly imprinted polymer poly(methacrylic acid)-based quartz crystal microbalance) [158]	DPV	0.73 ng/mL/ 15.2–750 ng/mL
	D-CNT@PPy@Pt NPs@beta-CD (polypyrrole-coated chiral carbon nanotubes with Pt nanoparticles and beta-cyclodextrin) [157]	CV	0.133 × 10^−9^/ 19.6–196 × 10^−6^	MIP-OECTs (molecularly imprinted polymer-organic electrochemical transistors) [149]	CV	2 × 10^−9^/ 11.6 and 3.5/ 300 × 10^−9^ to 10 × 10^−6^
	L/D-DHCNT@PPy@AuNPs@L/D-Cys (left/right-handed double helix carbon nanotubes/Polypyrrole@Au nanoparticles [156]	DPV	0.012 L-Trp% and 0.14 D-Trp%/ 0.659 and 0.02			

^1^ CA, chronoamperometry.
